# Mitotic Spindle Defects and Chromosome Mis-Segregation Induced by LDL/Cholesterol—Implications for Niemann-Pick C1, Alzheimer’s Disease, and Atherosclerosis

**DOI:** 10.1371/journal.pone.0060718

**Published:** 2013-04-12

**Authors:** Antoneta Granic, Huntington Potter

**Affiliations:** 1 Department of Neurology and Linda Crnic Institute for Down Syndrome, University of Colorado School of Medicine, Aurora, Colorado, United States of America; 2 Institute for Ageing and Health, Campus for Ageing and Vitality, Newcastle University, Newcastle upon Tyne, United Kingdom; Massachusetts General Hospital/Harvard Medical School, United States of America

## Abstract

Elevated low-density lipoprotein (LDL)-cholesterol is a risk factor for both Alzheimer’s disease (AD) and Atherosclerosis (CVD), suggesting a common lipid-sensitive step in their pathogenesis. Previous results show that AD and CVD also share a cell cycle defect: chromosome instability and up to 30% aneuploidy–in neurons and other cells in AD and in smooth muscle cells in atherosclerotic plaques in CVD. Indeed, specific degeneration of aneuploid neurons accounts for 90% of neuronal loss in AD brain, indicating that aneuploidy underlies AD neurodegeneration. Cell/mouse models of AD develop similar aneuploidy through amyloid-beta (Aß) inhibition of specific microtubule motors and consequent disruption of mitotic spindles. Here we tested the hypothesis that, like upregulated Aß, elevated LDL/cholesterol and altered intracellular cholesterol homeostasis also causes chromosomal instability. Specifically we found that: 1) high dietary cholesterol induces aneuploidy in mice, satisfying the hypothesis’ first prediction, 2) Niemann-Pick C1 patients accumulate aneuploid fibroblasts, neurons, and glia, demonstrating a similar aneugenic effect of intracellular cholesterol accumulation in humans 3) oxidized LDL, LDL, and cholesterol, but not high-density lipoprotein (HDL), induce chromosome mis-segregation and aneuploidy in cultured cells, including neuronal precursors, indicating that LDL/cholesterol directly affects the cell cycle, 4) LDL-induced aneuploidy requires the LDL receptor, but not Aß, showing that LDL works differently than Aß, with the same end result, 5) cholesterol treatment disrupts the structure of the mitotic spindle, providing a cell biological mechanism for its aneugenic activity, and 6) ethanol or calcium chelation attenuates lipoprotein-induced chromosome mis-segregation, providing molecular insights into cholesterol’s aneugenic mechanism, specifically through its rigidifying effect on the cell membrane, and potentially explaining why ethanol consumption reduces the risk of developing atherosclerosis or AD. These results suggest a novel, cell cycle mechanism by which aberrant cholesterol homeostasis promotes neurodegeneration and atherosclerosis by disrupting chromosome segregation and potentially other aspects of microtubule physiology.

## Introduction

High levels of dietary cholesterol and plasma LDL have been found to constitute a common risk factor for both atherosclerosis/cardiovascular disease and for Alzheimer’s disease, but the mechanism(s) of this effect are incompletely understood [1], [2]. Atherosclerosis is characterized by localized accumulations of lipids, inflammatory cells, smooth muscle cells and calcified cell debris [3], while Alzheimer’s disease (AD) is characterized by aberrant oligomerization/polymerization of two misfolded proteins–extracellular amyloid-beta (Aß) assembled into amyloid deposits, and intracellular hyperphosphorylated tau assembled into neurofibrillary tangles [4]–[6]. We sought to determine whether there is a common pathogenic pathway by which cholesterol/LDL promotes the development of both atherosclerosis and Alzheimer’s disease.

Genetic, biochemical, and transgenic mice studies of mutations that cause familial forms of AD (FAD) have identified the Aß peptide as central to AD pathogenesis, with Apolipoprotein E (ApoE), Tau, and microtubules being required for Aß to oligomerize/polymerize and induce synaptic loss, neurodegeneration, and dementia [4]–[7].

### Cell Cycle Defects and Chromosome Mis-segregation in AD

One mechanism by which Aß evidently causes neurodegeneration is by interfering with cell cycle. For example, we proposed that AD subjects would exhibit chromosome mis-segregation and the accumulation of aneuploid, particularly trisomy 21 cells and then used primary skin fibroblasts to demonstrate such trisomy 21 mosaicism and a trend toward chromosome 18 mosaicism in both familial and sporadic AD (SAD) patients [8]–[11]. AD lymphocytes also showed trisomy 21 mosaicism and premature centromere division, a mechanistic precursor to chromosome mis-segregation [9], [12]–[16]. Work from several laboratories confirmed and extended these results to buccal cells and brain neurons, with trisomy 21 constituting 10% of neurons in late stage AD brain [17]–[21].

We investigated the mechanism of this cell cycle defect and found that mutations in the presenilin 1 (*PS1*) and amyloid precursor protein (*APP*) genes that cause FAD directly induce chromosome mis-segregation and up to 20% aneuploidy in lymphocytes and neurons of transgenic mice and in transfected cells. Indeed the Alzheimer Aß peptide, the proteolytic product of the PS1 based γ-secretase enzyme on the APP protein and the key player in AD pathogenesis, also induces abnormal spindle structure and chromosome mis-segregation, including human trisomy 21 and mouse trisomy 16 in transgenic mice, transfected cells, and cell-free Xenopus egg extracts [22]–[24], [25]–[28]. We found that his profound defect in mitosis in AD results from Aß inhibition of certain mitotic kinesin motor proteins, including kinesin5/Eg5, that are essential for the structure and function of the mitotic spindle [24].

That chromosome mis-segregation and the consequent development of aneuploidy plays an essential role in AD pathogenesis is indicated by the finding that following the development of 30% aneuploid neurons during the early stages of AD, the specific loss of these aneuploid neurons in the transition from mild cognitive impairment to late stage AD can account for 90% of the neuronal cell loss observed at autopsy [21].

The finding of trisomy 21 mosaicism in AD is particularly interesting because of the universal presence of AD-like pathology and neurodegeneration in full trisomy 21 Down syndrome (DS) patients [8], [29]–[31], and the finding that early onset inherited AD can be caused by a single duplicated *APP* gene on one chromosome 21 [32], [33]. Evidently a 50% excess of APP and its product Aß are sufficient for the development of AD. Case studies of patients with trisomy 21 mosaicism and no intellectual impairments of the DS type who developed AD by age 40 demonstrates that a small percentage of trisomy 21 cells can, over a lifetime, lead to and/or contribute to the pathogenesis of AD [8], [25], [34]–[37].

### Disruption of Cholesterol Homeostasis in Neurodegenerative Diseases

Because the generation and loss of aneuploid neurons appears to underlie the majority of AD neurodegeneration, it is essential to understand the mechanism by which such aneuploidy arises during the preclinical stage of the disease. Clearly, the overproduction of the Aß peptide may be partly responsible. However, defects in cholesterol homeostasis may also play a role in AD pathogenesis [38]–[43]. For example AD, and cardiovascular disease (CVD) share risk factors (e.g., high plasma cholesterol and a high cholesterol and/or saturated fat diet), and the severity of atherosclerotic lesions in AD brains correlates with the extent of AD pathology [38], [40]. Likewise, FAD transgenic mice fed a high cholesterol diet develop accelerated amyloid burden and steeper cognitive decline compared to animals on regular chow [39], [43]. Conversely, inhibition of endogenous cholesterol synthesis with statins has been associated with a reduced risk of AD and decreased amyloid deposits in humans and animals [42]. Finally, ApoE is the major cholesterol transporter in the brain, and inheritance of the *ApoE ε4* allele is the strongest genetic risk factor for sporadic AD [44]. ApoE particularly ApoE4 directly associates with Aß and catalyzes its polymerization into neurotoxic assemblies [7].

A dysregulation of cholesterol homeostasis has also been implicated in other chronic neurodegenerative diseases, including Niemann-Pick (NPC), Huntington, and Parkinson’s disease [39], [43]. Specifically, a direct association between disrupted intracellular cholesterol trafficking and consequent neuronal loss, gliosis, and formation of neurofibrillary tangles (NFT) in individuals carrying a mutation in the *NPC1* or *NPC2* gene has been established. Loss-of function mutations in these genes cause sequestration of unesterified cholesterol in late endosomes/lysosomes in a number of cells and tissues, but are most deleterious for brain development and health, triggering progressive neuropathology, ataxia, dementia and premature death in early adolescence [43]. Recent studies utilizing human samples and cellular and animal models of NPC revealed interesting parallels between AD and NPC neuropathogenesis, which include endosomal/lysosomal dysfunction, glial-mediated inflammation, NFTs and Aß accumulation especially in *ApoE ε4* carriers, cholesterol dyshomeostasis, and cell cycle reactivation [27], [28], [39], [43]. However, unlike AD [8]–[21], the presence of chromosomal abnormalities such as aneuploidy in peripheral and brain cells has not been reported in NPC.

These pathological correlations and our finding that Aß induces chromosome mis-segregation prompted us to investigate whether cholesterol might also induce aneuploidy in AD and possibly NPC and atherosclerosis. The latter hypothesis was reinforced by reports that each atherosclerotic plaque harbors a monoclonal proliferation of aneuploid smooth muscle cells [45]–[52].

To test these hypotheses, we asked whether cholesterol and lipoproteins induce chromosomal mis-segregation *in vivo* and *in vitro* and then investigated the mechanism of their observed novel aneugenic effect.

## Results

**Figure 1:**
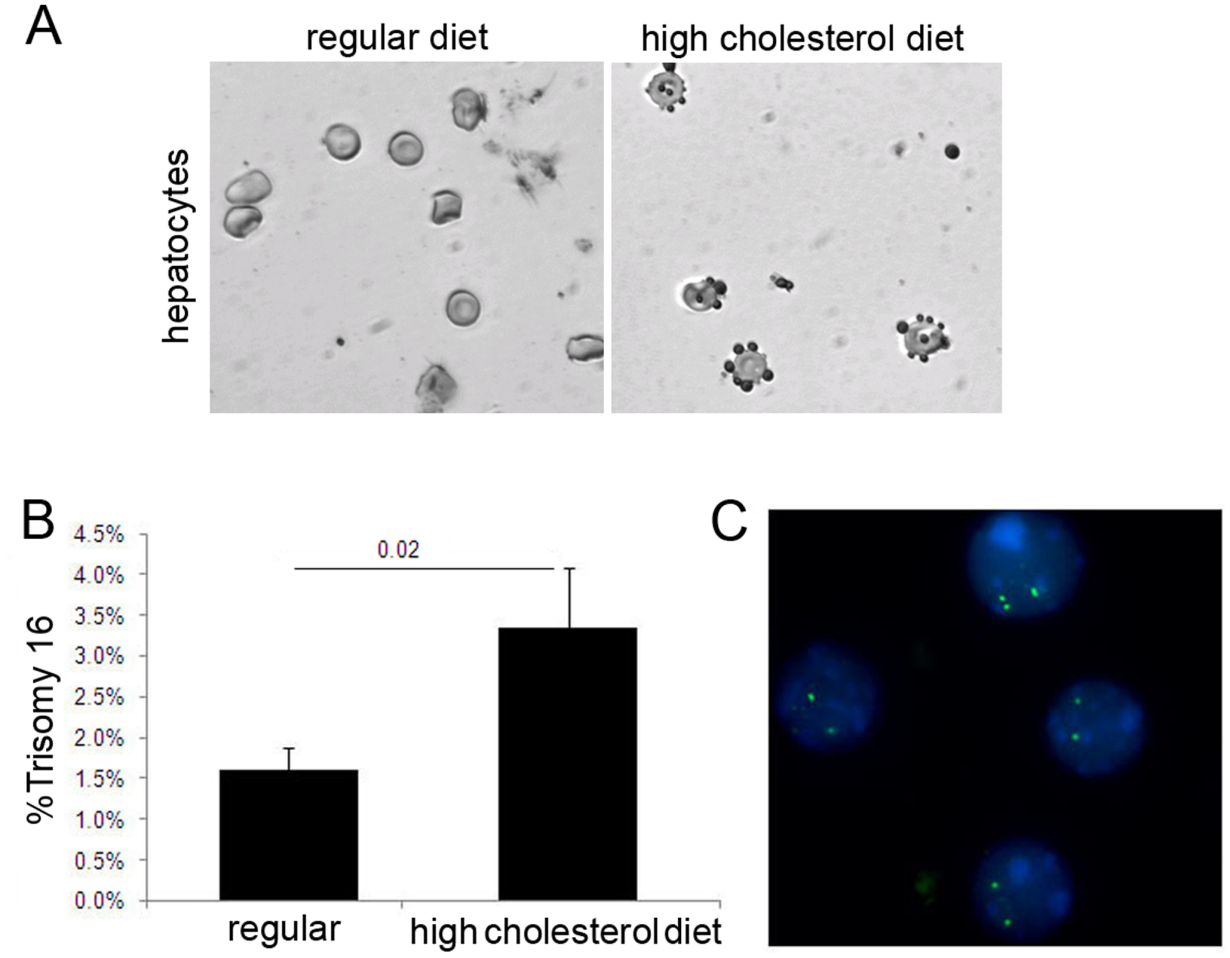
Increased cholesterol induces chromosome aneuploidy in vivo. (A) Mouse hepatocytes (hematoxylin blue stain; light gray in micrograph) were stained with Oil-Red-O stain (red stain, dark gray in micrograph) to detect accumulation of lipid droplets, a sign of liver steatosis and a consequence of dyslipidemia in mice fed a high cholesterol diet (right panel). (B–C) Quantitative FISH analysis for chromosome 16 showed that young wild-type mice fed a high (1.05%) cholesterol, atherogenic diet for 12 weeks developed higher levels of trisomy 16 in spleen cells compare to mice fed regular chow.

**Figure 2:**
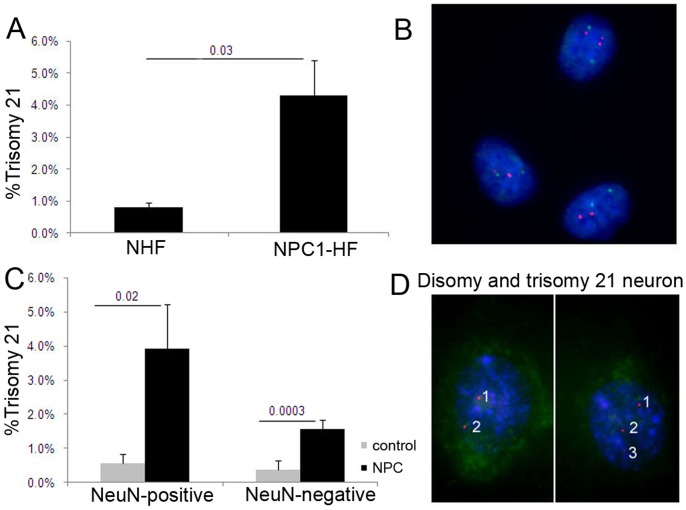
Increased trisomy 21 aneuploidy in fibroblasts and in glia and neurons of Niemann-Pick C1 patients. (A,B) FISH analysis with a DNA probe for chromosome 21 (red) and chromosome 12 (green) of fibroblasts derived from NPC1 patients (NPC1-HF) showed an increase in trisomy 21 cells compared to age-matched normal human fibroblasts. (C,D) Quantitative FISH analysis with a DNA probe for chromosome 21 (red) followed by staining with NeuN antibody (green) and DAPI (blue) of resuspended cells from frontal cortices of control and NPC brains revealed significantly higher levels of trisomy 21 in NPC neurons and glia compared to controls.

**Figure 3:**
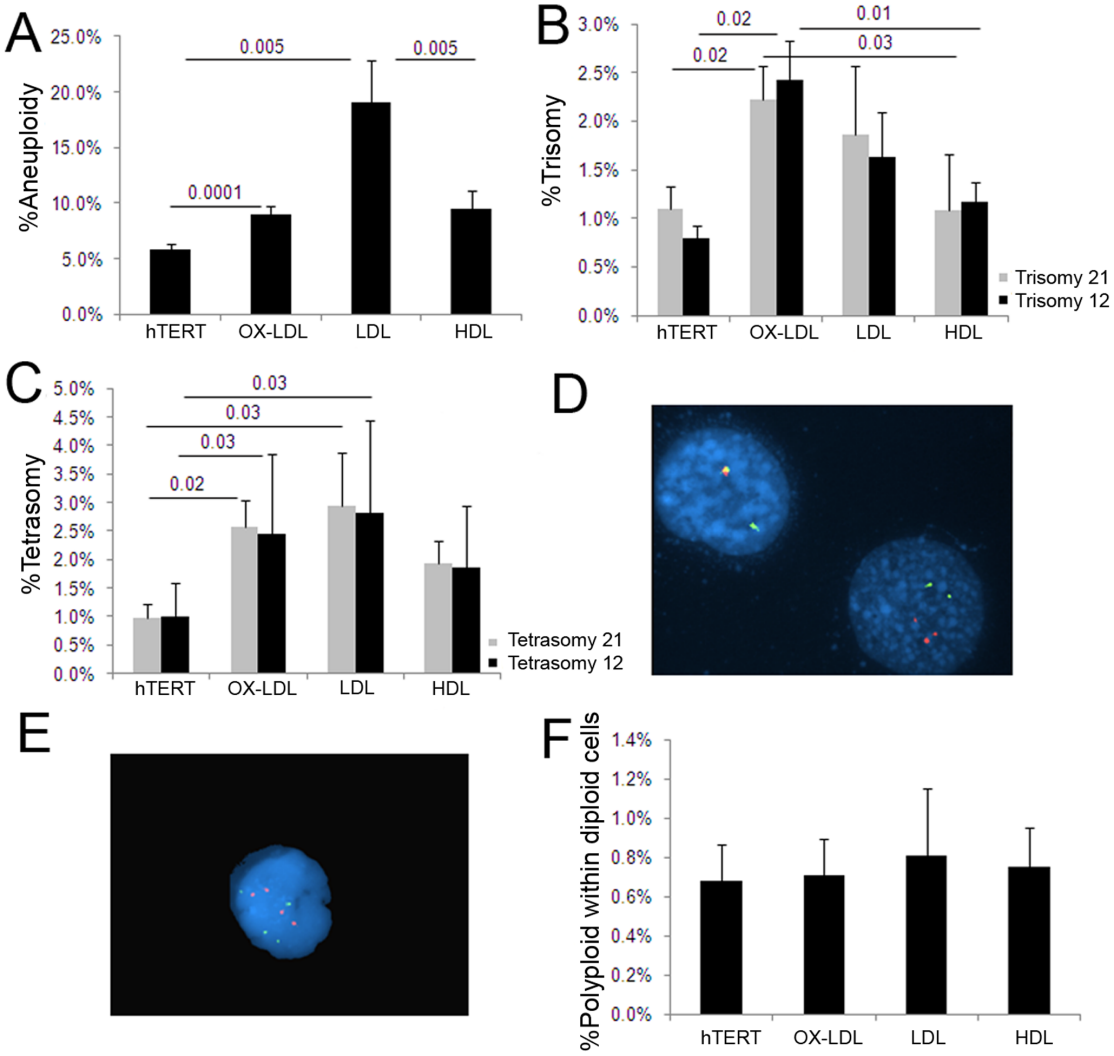
Lipoprotein treatment induces aneuploidy. (A) Actively growing hTERT-HME1 cells were treated with 20 µg/ml of OX-LDL, LDL or HDL for 48 hr, arrested in metaphase and Giemsa stained for karyotype analysis. All lipids induced significantly higher levels of aneuploidy compared to untreated cells, with LDL and or OX-LDL exhibiting a much stronger aneugenic effect than HDL. (B–E) FISH analysis of the same lipoprotein-treated cells showed that OX-LDL-induced trisomy 21 and trisomy 12 (B,D), and that both OX-LDL and LDL-induced tetrasomy 21 and 12 (C,E). (F) Karyotype analysis of an aliquot of the cells from the same treatment showed very few, but equal numbers of polyploid cells, indicating that the tetrasomies observed are due to chromosome mis-segregation of chromosomes 21 or 12, and not a result of chromosome duplication before cell division.

**Figure 4:**
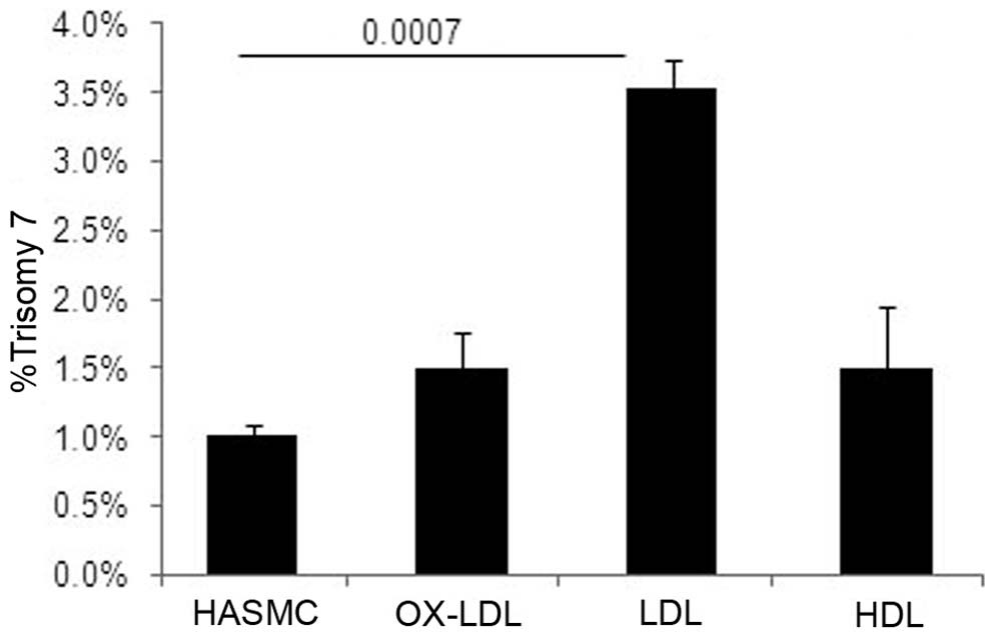
LDL induces trisomy 7 in human aortic smooth muscle cells. Quantitative FISH analysis of HASM cells showed an increase in trisomy 7 when incubated with 20 µg/ml of LDL, but not HDL for 48 hr.

**Figure 5:**
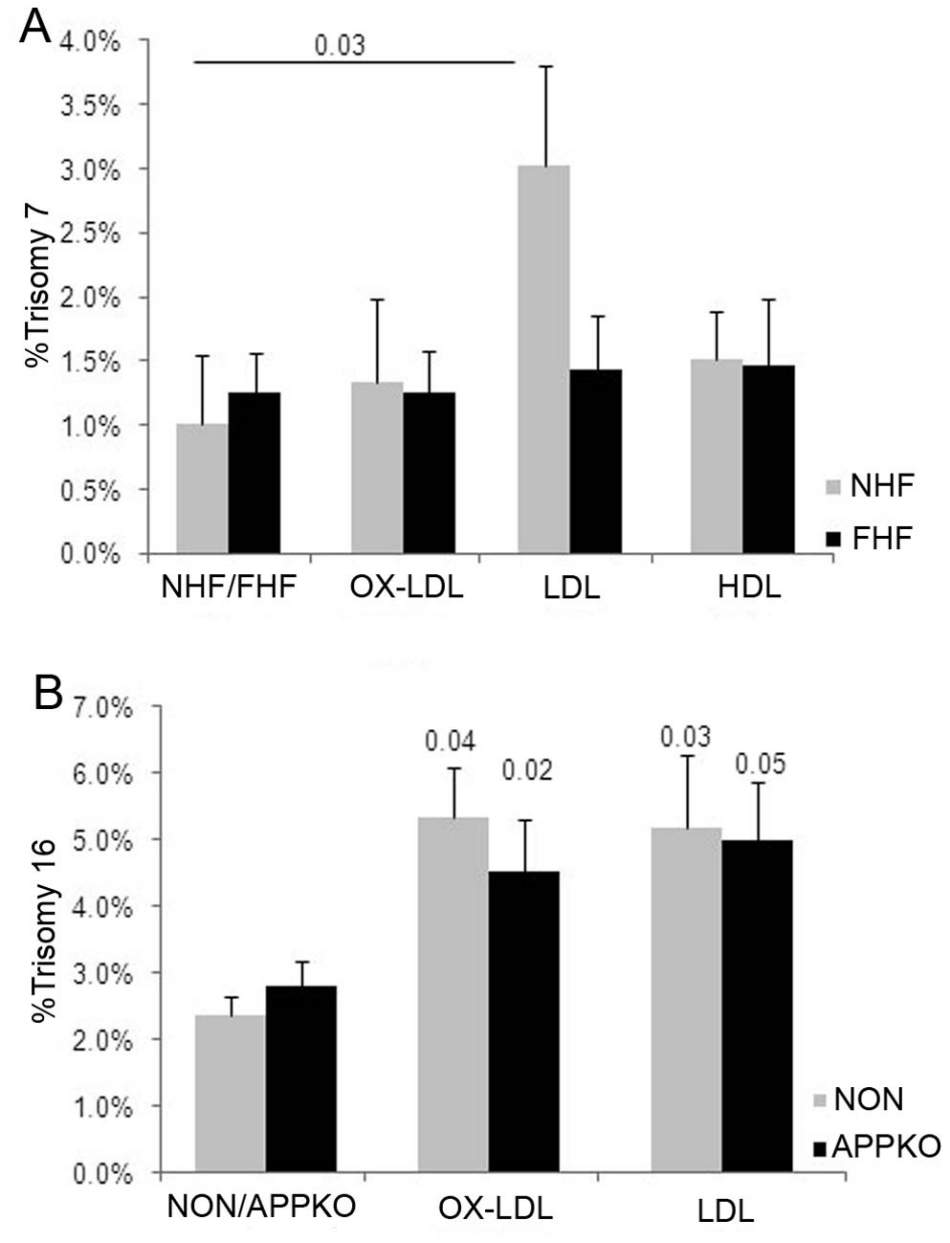
Lipoprotein-induced aneuploidy is dependent on LDLR and independent of APP. (A) Normal human fibroblasts (NHF) developed significantly higher levels of trisomy 7 when treated with 20 µg/ml of LDL for 48 hr compared to LDL receptor deficient human fibroblasts obtained from the patient diagnosed with familial hypercholesterolemia (FHF). (B) Quantitative FISH for chromosome 16 revealed comparable levels of trisomy 16 in primary splenocytes derived from nontransgenic (NON) and APP knockout (APPKO) mice upon incubation with 20 µg/ml of OX-LDL or LDL for 48 hr, indicating that the aneugenic activity of lipoproteins is independent of a functional APP gene and of its product Aβ.

**Figure 6:**
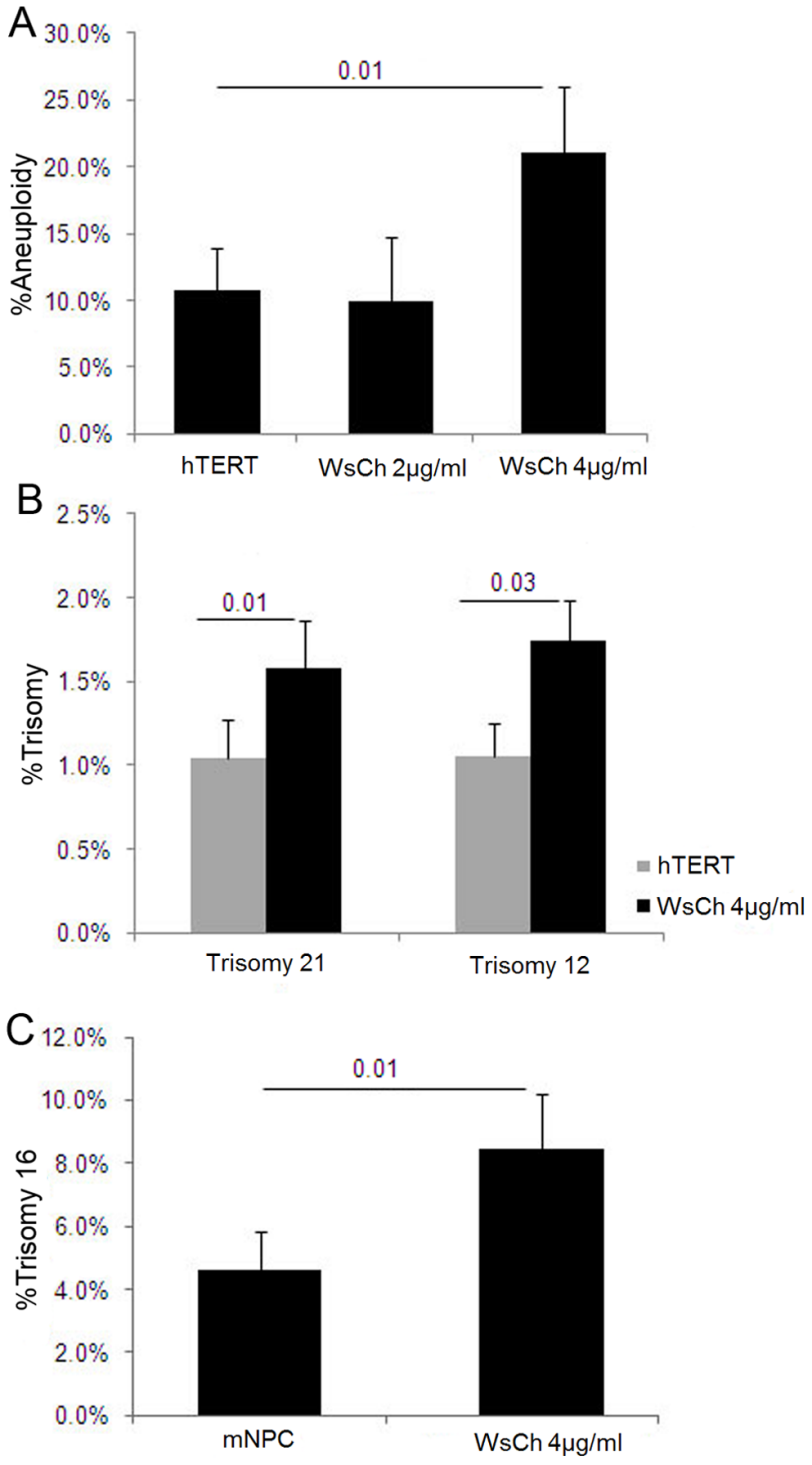
Increased membrane cholesterol induces aneuploidy. (A,B) Karyotype and FISH analysis showed an increase in total aneuploidy (A) and trisomy 21 and trisomy 12 (B) in hTERT cells treated for 48 hr with 4 µg/ml cholesterol made water-soluble in a methyl-β-cyclodextrin (MβCD) complex. (C) Mouse chromosome 16 DNA probe was used to measure aneuploidy levels in mouse neuronal precursor cells (mNPC) derived from prenatal brains of wild-type mice (E17–18) and incubated with and without 4 µg/ml of WsCh for 7 days. Quantitative FISH analysis showed a 2–fold increase in trisomy 16 in WsCh-treated cells compared to controls. mNPC harbored up to 4.6% endogenous aneuploidy, as reported previously in developing mouse and human brains [73].

**Figure 7:**
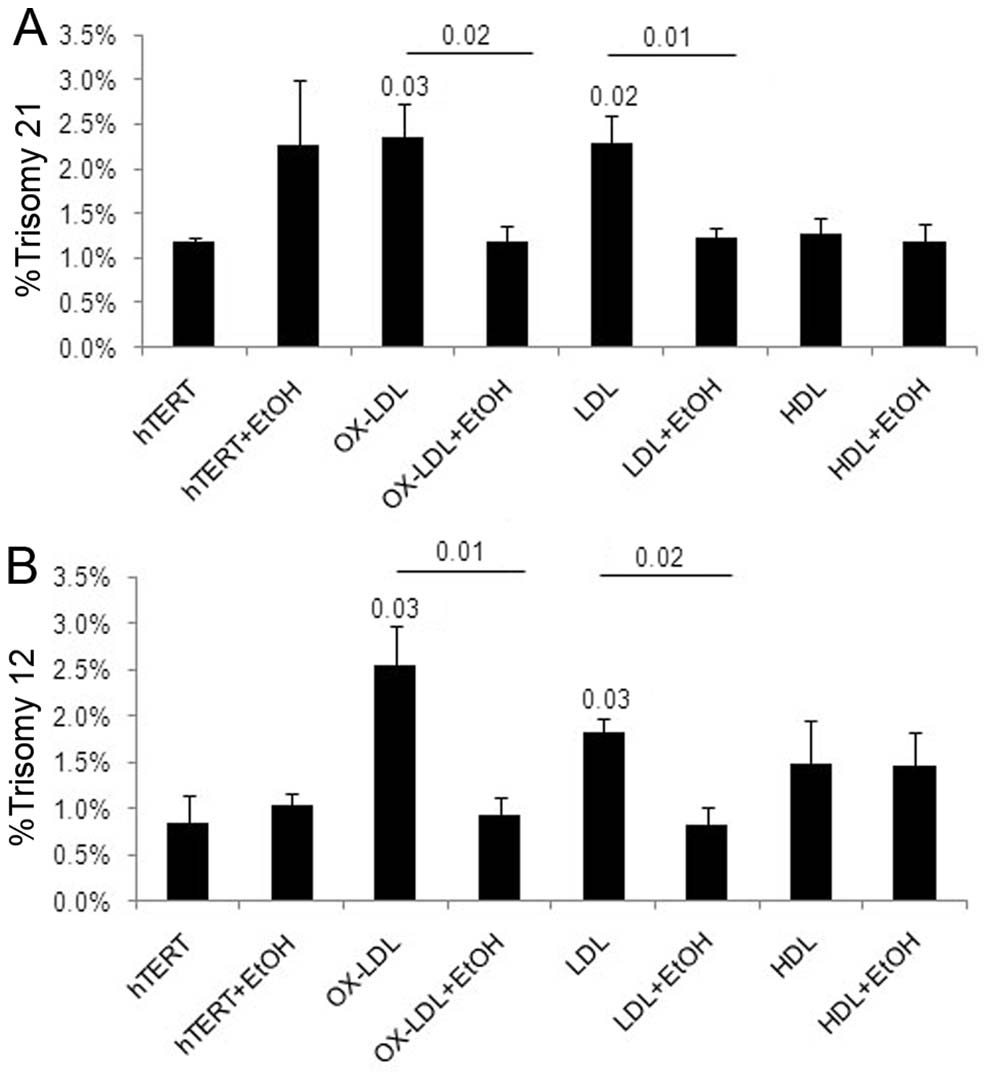
Ethanol attenuates lipoprotein-induced chromosome mis-segregation. (A,B) Quantitative FISH analysis of hTERT cells pre-treated with 25 mM of ethanol (EtOH) for 24 hr and co-incubated with lipoproteins and EtOH for an additional 48 hr revealed a decrease in OX-LDL and LDL-induced trisomy 21 (A) and trisomy 12 (B).

**Figure 8:**
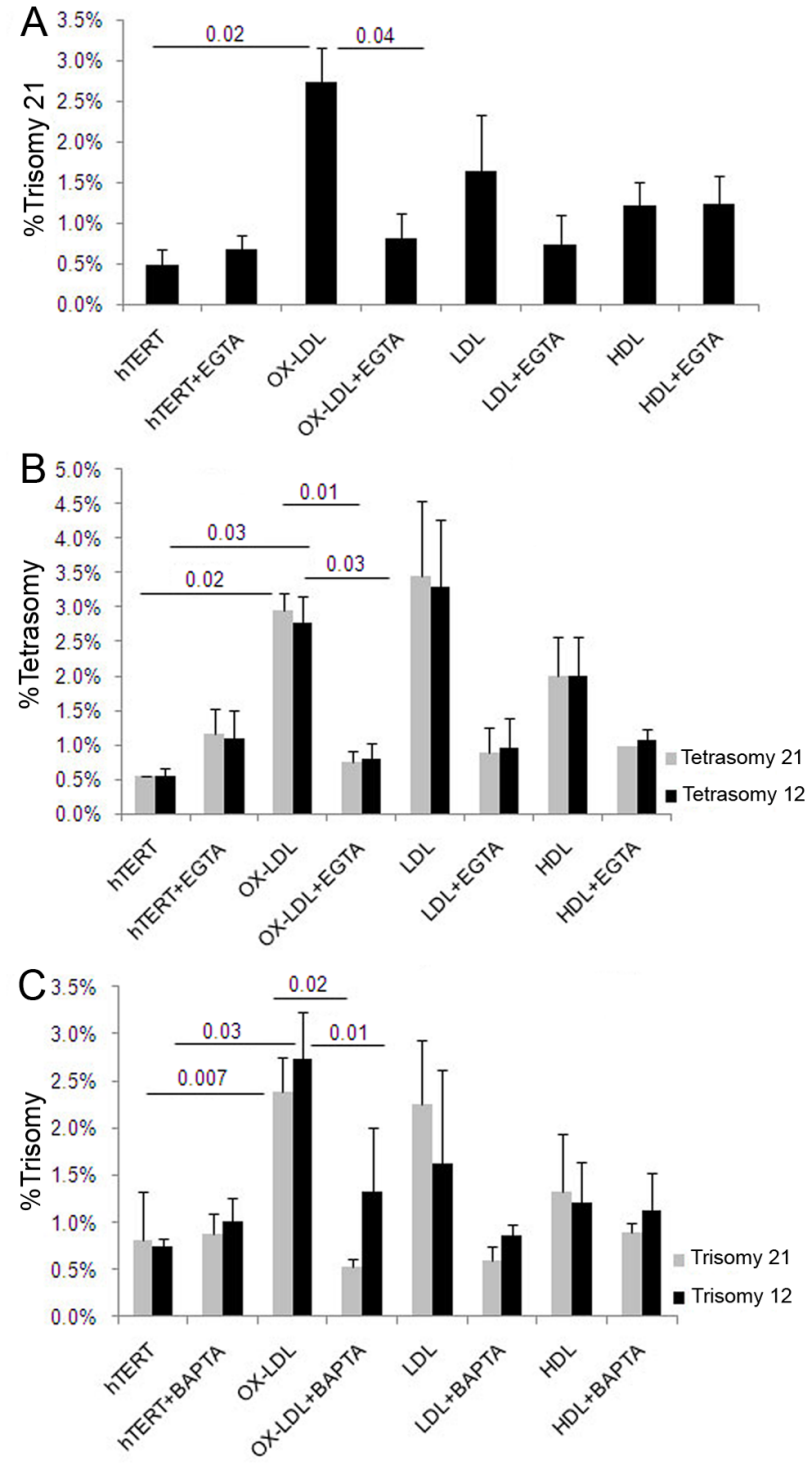
Role for Ca++ in lipoprotein induced chromosome mis-segregation. (A–C) Quantitative FISH analysis with a dually labeled DNA probe for chromosomes 21 and 12 showed a significant reduction in trisomy 21 (A), tetrasomy 21 and tetrasomy 12 (B) in hTERT cells pre-treated with 1.5 mM of Ca++ chelator EGTA followed by OX-LDL compared to cells treated only with OX-LDL. Cells pre-treated with 1 mM BAPTA had significantly lower levels of OX-LDL-induced trisomy 21 and trisomy 12 (C) compared to the cells grown without chelator.

**Figure 9:**
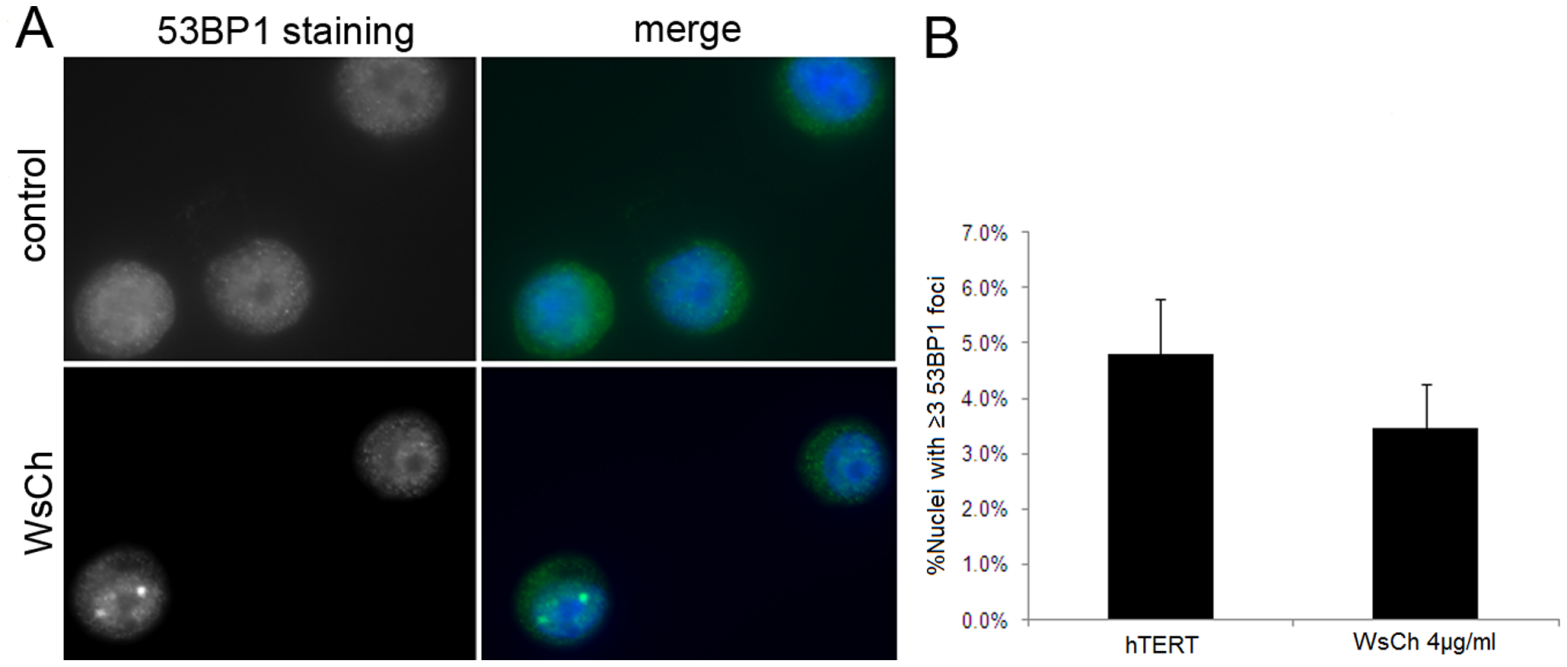
Cholesterol does not cause DSBs in hTERT-HM1 cells. (A) hTERT-HME1 cells were exposed to 4 µg/ml of cholesterol for 48 hr and immunostained for a double-strand breaks (DSBs) marker, the p53 binding protein 53 BP1. Immunofluorescent 53 BP1 (green) foci in nuclei (DAPI, blue) of control and cholesterol-treated cells were counted. (B) There was no statistically significant (p = 0.06) increase in DSBs events (≥3 foci per nucleus) in cholesterol-treated compared to untreated cells. The nuclei containing ≥4 foci were extremely rare.

**Figure 10:**
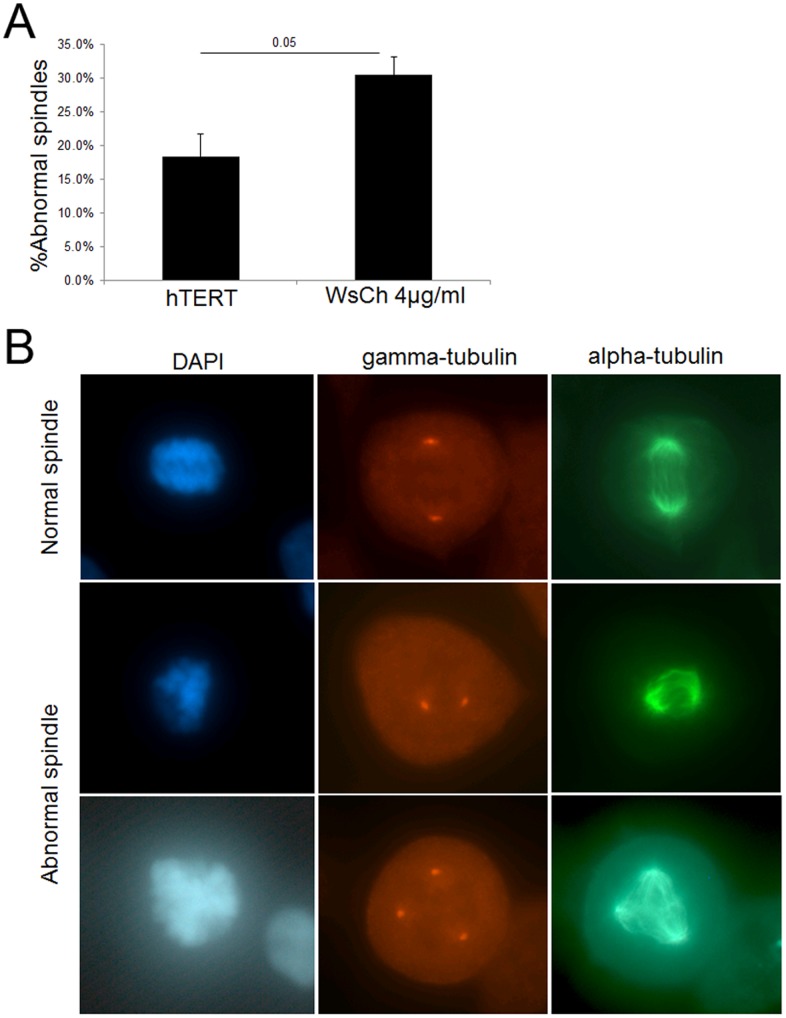
Mitotic spindle structure disrupted by cholesterol. (A) hTERT cells were treated for 24 hr with 4 µg/ml WsCh and the structure of the mitotic spindles observed and analyzed for abnormal DNA localization, lagging chromosomes, super-numerary centrosomes, and dis-arrayed microtubules. There was a significant increase in abnormal mitotic spindle structure induce by cholesterol exposure, with dis-arrayed microtubules and mis-localized DNA being the most prominent defects. (B) Examples of normal spindles in untreated cells and abnormal spindles in cholesterol-treated cells.

To investigate whether lipoproteins and cholesterol affect chromosome segregation, we approached the project in three phases. First, metaphase chromosome analysis and fluorescence *in situ* DNA hybridization (FISH) were used to determine whether LDL/cholesterol increases the levels of total and chromosome specific aneuploidy *in vivo*: in mice fed a high cholesterol diet and in human Niemann-Pick patients with a mutation in the *NPC1* gene, which is implicated in cholesterol trafficking and atherosclerosis but has not previously been associated with a defect in chromosome segregation, although mitosis-specific epitopes have been observed in NCP1 brains [53], [54] (Figure 1,2). Then, to establish that lipoproteins/cholesterol were directly responsible for the aneuploidy we observed in cholesterol-fed mice and NPC1 patients, we analyzed the chromosomes of different cells in culture after exposure to various lipids, including water solubilized cholesterol (Figures 3–4). Particular attention was paid to assessing the aneuploidy of chromosomes 7, 12, 14, 18 and 21, as mis-segregation of several of these chromosomes has been found in AD [9], [11]–[23] and in atherosclerotic lesions [46]–[51]. We then investigated the mechanism by which lipoproteins/cholesterol induce chromosome mis-segregation by analyzing their effect in cells lacking the low density lipoprotein receptor (*LDLR*) or the *APP* gene or exposed to calcium chelators or ethanol to counter the intracellular signaling and the membrane rigidifying effect of cholesterol (Figures 5–8). Finally we determined whether cholesterol treatment induces DNA damage (Figure 9) or disrupts the mitotic spindle, for example leading to multiple centrosomes (MOCs), lagging chromosomes and mis-aligned/displaced DNA or disarrayed microtubules, all of which are known causes of chromosome mis-segregation/instability (Figure 10).

### Cholesterol Dyshomeostasis Induces Aneuploidy *in vivo*

#### Mice fed a high cholesterol diet develop aneuploidy in splenocytes

The effect of hypercholesterolemia on chromosome-segregation in peripheral cells (T-splenocytes) and in brain cells was assessed in atherosclerosis-susceptible wild-type mice, the C57BL/6 strain [55]. One-month-old (nontransgenic) mice (4 males and 4 females) were randomly assigned to normal mouse chow (0% cholesterol) or a high cholesterol (∼1.05%) diet (two mouse pairs per group) for 12 weeks. Mice fed the regular diet consumed on average 50.62±8.34 g/mouse pair/week (∼172 Kcal of Digestible Energy (DE)/mouse pair/week), while mice on the high cholesterol diet consumed on average 50.83±11.14 g/mouse pair/week (∼229 Kcal of DE/mouse pair/week). Because of the difference in caloric value between the diets, mice on the high cholesterol diet gained on average 2½ times more weight than mice fed regular chow (p<0.05). Pathological examination at sacrifice confirmed that only mice on the high cholesterol diet developed hepatic steatosis, a sign of dyslipidemia, which was confirmed with Oil-Red-O staining of isolated hepatocytes (Figure 1A).

Primary splenocyte cultures and brain cell suspensions from mice on the regular and atherogenic diets were analyzed for aneuploidy. FISH analysis of concanavalin A-stimulated spleen T-cells from mice fed the high cholesterol diet showed a significant increase in trisomy 16 compared to T-splenocytes of mice fed the regular diet (Fig. 1B,C), but there was no induction of aneuploidy in brain cells (0.76% versus 0.65%, p = 0.3). The fact that diet-induced hypercholesterolemia has not been reported to affect brain cholesterol homeostasis in wild-type mice suggests that serum cholesterol may have induced the spleen cell aneuploidy while sparing the brain. It is also possible that 12 weeks is sufficient time to induce the development of aneuploid cells in mitotically active peripheral (lymphatic) tissues of animals fed cholesterol, but is insufficient to allow accumulation of significant aneuploidy in the brain resulting from relatively less active neurogenesis.

#### Niemann-Pick (*NPC1*) mutant neurons, glia, and fibroblasts accumulate elevated levels of trisomy 21

To investigate the role of disrupted peripheral and central cholesterol homeostasis in chromosome segregation in a longer-term human neurodegenerative disease model, we turned to primary cells derived from patients diagnosed with Niemann-Pick Type C (NPC) disease (Table 1). Mutations and deletions in the *NPC1* and *NPC2* genes cause impaired trafficking of unesterified cholesterol (both low density lipoprotein receptor (LDLR)-internalized and endogenously synthesized) and other lipids (e.g., glycosphingolipids), which accumulate in late endosomes and lysosomes and fail to travel to the plasma membrane and endoplasmic reticulum (ER), resulting in severe cognitive deficits [56], [57]. Loss/defect in the *NPC1* gene has also been shown to promote atherosclerosis in animal models [58]. Interestingly, as was first reported in AD, mitosis-specific proteins and/or phospho-epitopes have been found to be upregulated in NPC1 brains [27], [28], [53], [54], [59]–[65].

**Table 1:** Characteristics of the Niemann-Pick Type C (NPC) and Control Samples.

Repository #	Age yr	Gender	Clinical Dx	Mutation of NPC assay
***Control fibroblasts***				
AG02101	27	female	none	N/A
AG02603	35	female	none	N/A
AG09319	24	female	none	N/A
AG09429	25	female	none	N/A
***NPC1 fibroblasts***				
GM18422	NK	female	NPC1	813_815delCAT & ASP874VAL
GM18390	NK	female	NPC1	ASP242HIS & SER940LEU
GM22871	4	female	NPC1	1920delG & IVS9–1009G>A
GM17912	19	female	NPC1	PRO1007ALA & THR1036MET
***Control brains***				
UMB754	11.5	female	asthma	N/A
UMB1864	2.5	female	streptococcus infection	N/A
UMB4725	32.2	female	hypertension	N/A
UMB55	19.6	female	auto accident injury	N/A
UMB4590	20.5	female	dilated cardiomyopathy	N/A
***NPC brains***				
UMB5372	11.3	female	NPC1	filipin & cholesterol esterification
UMB4214	32.3	female	NPC	confirmed NPC neuropathology
UMB4237	19.8	female	NPC	confirmed NPC neuropathology
UMBM4992M	20.1	female	NPC	NPC clinical diagnosis

yr = year; NK = not known. Human fibroblasts were purchased from Coriell and brains from NICHD Brain and Tissue Bank for Developmental Disorders. Independent T-test revealed no age difference between NPC and control brain donors (20.9±8.6 vs. 17.3±11.1, p = 0.61).

To test whether cholesterol mal-distribution affects chromosome stability in NPC disease, we assessed the level of aneuploidy in primary NPC fibroblasts and brain cells hybridized with FISH probes for chromosomes 12, 21, 14 and/or 18. We observed a 4-fold increase in trisomy 21 in NPC1 fibroblasts (Figure 2A,B) compared to normal fibroblasts and an increase of aneuploidy 12 that did not reach statistical significance (2.1% versus 0.4%, p = 0.12). Quantitative FISH analysis of Neu-N-positive and Neu-N-negative brain cells showed 4 to 6-fold increases in trisomy 21 in both neurons and glia with the *NPC1* mutation, compared to the cells from control brains (Fig. 2C,D). However, there was no increase in aneuploidy of chromosomes 12, 14 or 18 in NPC brain cells compared to controls (0.55% vs. 0.24%, p = 0.11; 1.61% vs. 1.46%, p = 0.41; and 0.75% vs. 0.78%, p = 0.45, respectively).

These results are the first demonstration of a genomic instability and trisomy 21 mosaicism in Niemann-Pick disease and indicate that a cholesterol accumulation/localization defect causes chromosome mis-segregation in humans, as a high cholesterol diet did in mice.

### Lipoprotein/cholesterol Induces Aneuploidy *in vitro*

#### hTERT-HME1 cells treated with lipoproteins develop aneuploidy

To determine whether the aneuploidy observed in NPC patients or in mice fed an atherogenic diet reflects a direct effect of lipids on mitosis, hTERT-HME1 cells were exposed to 20 µg/ml oxidized LDL (OX-LDL), LDL or high density lipoprotein (HDL) for 48 hr and assessed for aneuploidy by metaphase chromosome analysis and FISH, as described. This lipid concentration was chosen to mimic the cholesterol level in human blood and to approximate tissue culture conditions. The results showed an up to 4-fold increase in abnormal chromosome complements (20% aneuploidy) in OX-LDL and LDL treated cells compared to controls and to HDL-treated cells (Figure 3A). The chromosome specific aneuploidy was further analyzed by quantitative FISH (Figure 3B–E), which revealed an induction of: 1) trisomy 21 and 12 by OX-LDL and a borderline significant induction of trisomy 21 and 12 by LDL (Figure 3B and D), and 2) induction of tetrasomy 21 and 12 by both OX-LDL and/or LDL (Figure 3C and E).

A small number of tetraploid neurons has been observed in AD brains of humans and mice and interpreted to be a consequence of aberrant cell cycle activation and DNA duplication without cell cycle completion [25], [27], [28], [59]–[65]. The OX-LDL and LDL induced tetrasomy we observed in interphase hTERT-HME1 cells are most consistent with severe chromosome mis-segregation because very few tetraploid/polyploid cells were observed in the metaphase spreads (Figure 3F), which was determined by counting a total number of metaphase hyperploid/polyploid cells (having at least ≥90 chromosomes) and diploid cells (46 chromosomes [2 n]) in the portion of the slide initially analyzed for karyotype. Also, individual cells with both tetrasomy 21 and 12 occur at similar levels in treated and untreated cells (Figure 3E). Similarly in AD brain, many (10X) fewer tetraploid (4 n) neurons are observed compared to aneuploid (>2 n and <4 n) neurons [21].

#### LDL- induced trisomy 7 in HASM cells

To investigate the potential role of elevated lipoproteins on chromosome segregation during atherosclerosis, we tested the aneugenic effect of LDL, OX-LDL, and HDL (20 µg/ml) for 48 hr on primary human aortic smooth muscle cells (HASMC), which are prone to trisomy 7 in atherosclerotic plaques. Quantitative FISH revealed a statistically significant 3-fold increase in trisomy 7 in LDL treated cells (Figure 4).

#### Requirement for *LDLR* and *APP* in LDL-induced chromosome mis-segregation

1. Lipoprotein induced aneuploidy is dependent of *LDLR.* The LDL receptor belongs to a large family of structurally and functionally related cell surface receptors that are involved in diverse cellular functions, some implicated in AD pathogenesis, including cholesterol uptake and metabolism, ApoE binding [66], and APP trafficking and processing [67], [68]. More than 1000 unique *LDLR* genetic alterations have been identified that lead to Familial Hypercholesterolemia (FH), causing three to four times higher levels of blood LDL cholesterol than normal and premature CVD events, including severe atherosclerosis [69].

To investigate whether lipoprotein-induced chromosome mis-segregation requires a functional LDLR, FH primary human fibroblasts harboring mutations in both *LDLR* alleles and normal human fibroblasts were treated with 20 µg/ml of lipoproteins for 48 hr and assessed for aneuploidy. Quantitative FISH analysis revealed that LDL-treated normal human fibroblasts develop significantly higher levels of trisomy 7 compared to LDL-treated FH fibroblasts (Figure 5A).

2. Lipoprotein induced aneuploidy is independent of *APP.* Previously we found that Aß-induced aneuploidy is dependent on endogenous APP, possibly as a cell surface receptor to aid APP endocytosis and generation of intracellular Aß peptide or for the uptake of extracellular Aß [23], [70], [71].

To determine whether lipoprotein/cholesterol and Aß-induced chromosome mis-segregation are mechanistically related, we isolated primary splenocytes from *APPKO* mice and nontransgenic littermates, exposed them to lipoproteins, and scored them for chromosome 16 aneuploidy (Figure 5B). OX-LDL and LDL induced significant increases in trisomy 16 in both cell types, indicating that the aneugenic effect of lipoproteins is independent of APP expression and Aß production.

### Lipids and Cholesterol-induced Aneuploidy and Spindle Defects are Mediated through Membrane Fluidity and Calcium Homeostasis

#### Water-Soluble Cholesterol (WsCh) induces aneuploidy *in vitro*

1. WsCh induces aneuploidy in hTERT-HME1 cells. Cholesterol, the most abundant lipid in eukaryotic cell membranes [72], is compartmentalized into microdomains (e.g., lipid rafts), where it is required for essential cellular functions and structure, including signal transduction and plasma membrane dynamics [73], [74].

As indicated above, LDLR is used by cells to internalize external lipoproteins and is clearly required for the aneugenic effect of LDL. Methyl-β-cyclodextrin, MβCD, as cholesterol acceptor has been used to modify cellular cholesterol content, and to aid the delivery of hydrophobic cholesterol into the plasma membrane and intracellular compartments by enclosing it into its hydrophobic cavity, bypassing the need for an LDL receptor [75], [76]. To directly examine the effect of cholesterol on chromosome segregation, we exposed hTERT-HME1 cells to a water-soluble MβCD:cholesterol complex (WsCh) and performed karyotype and FISH analysis. First, we conducted a set of preliminary experiments with the concentrations of 2 to 10 µg/ml of WsCh for 24 and 48 hr of incubation to determine the optimal concentration at which aneuploidy induction could be measured over the background, but no visible morphological changes or cytotoxicity could be detected. 4 µg/ml of cholesterol exposure (but not 2 µg/ml) for 48 hr induced a 2-fold increase in total aneuploidy compared to untreated cells (Figure 6A) and a significant increase in trisomy 21 and trisomy 12 (Figure 6B), without altering cell proliferation (p = 0.25). Taken together, these data indicate that adding cholesterol to cell membranes induces mitotic defects in the form of aneuploidy likely affecting all chromosomes.

2. WsCh induces trisomy 16 in mouse neuronal precursor cells. Induction of chromosome mis-segregation by altered membrane cholesterol in AD or NPC patients is likely to be especially detrimental during neurogenesis and/or neuro-regeneration.

To investigate the effect of cholesterol on the development of aneuploidy in neuronal precursor cells, we prepared neurospheres from prenatal brains of nontransgenic mice, treated them with WsCh, and performed FISH for chromosome 16 as described. Parallel cultures were incubated with or without WsCh for 7 days (equivalent to the two cell divisions used previously for the hTERT-HME1 cells), with fresh cholesterol-containing media being replenished every other day to allow for expansion of the spheres, a sign of neuronal precursor cell proliferation [77]. We observed >4% trisomy 16 in untreated cells (Figure 6C), as has been previously reported in developing and adult normal human and mouse brains [78]–[80] and a significant 2-fold increase in trisomy 16 in cholesterol treated cells (Figure 6C).

#### Changing membrane fluidity by ethanol attenuates lipoprotein-induced chromosome mis-segregation

The fact that the addition of cholesterol to cells induces chromosome mis-segregation is a potentially important indicator of its underlying mechanism of action. It is well established that membrane cholesterol content is directly proportional to membrane stiffness/rigidity [81], which can be increased by cholesterol enrichment or decreased by cholesterol depletion [82]. In contrast, ethanol (EtOH) increases membrane fluidity, with profound physiological effects on multiple mammalian cells, including neurons [83]. Because both lipoprotein- and MβCD-delivered cholesterol increases membrane cholesterol content and rigidity *in vitro* [81], [82], [84], we hypothesized that the mitotic errors induced in cells by lipoprotein/cholesterol might be caused by increased membrane rigidity, which could be counteracted by the membrane fluidizing action of ethanol.

hTERT-HME1 cells were pre-treated with 25 mM of EtOH for 24 hr and further co-incubated with lipids in EtOH-containing media. The rationales for using this concentration of EtOH were: 1) 25 mM or 0.115% corresponds to the blood alcohol levels of moderate drinkers (1–3 drinks a day, 15–45 g) which has been associated with a decrease in risk for CVD [85], [86] and AD [87], 2) increased cholesterol efflux and cholesterol transport but unchanged cholesterol synthesis have been observed in human astrocytes *in vitro* at a concentration of 25 mM of EtOH in the presence and absence of cholesterol acceptors [88], 3) 25 mM EtOH fluidized the exofacial leaflet of the synaptic membranes [89], 4) long-term exposure of hepatic cells to 0.5% EtOH had no effect on LDLR expression or the uptake of LDL [90], and 5) our pilot experiments showed low cytotoxicity and up to ∼95% cell viability after 48 hr of 25 mM EtOH exposure and only a small, non-significant induction of aneuploidy for chromosome 21 (HSA21) (Figure S1A–D).

Quantitative FISH analysis of lipoprotein/EtOH-treated cells showed a significant decrease in OX-LDL and LDL-induced trisomy 21 (Figure 7A), and trisomy 12 (Figure 7B) compared to cells exposed only to lipoproteins, indicating that fluidization of the membranes by EtOH stabilizes chromosome segregation upon lipid treatment.

#### Extracellular Ca++ is required for lipoprotein-induced chromosome mis-segregation

It has been postulated that perturbed calcium homeostasis plays an important role during aging and in AD pathogenesis, and calcium is essential for the function of many enzymes, for example CamKII [91]. In our recent study of Aß-induced aneuploidy, we showed that chelation of extracellular Ca++ with BAPTA prevents chromosome mis-segregation *in vitro* [23].

To elucidate whether Ca++ plays a role in lipoprotein/cholesterol-induced aneuploidy, as it does in Aß-induced aneuploidy, we conducted experiments with hTERT-HME1 cells using two different chelating reagents, EGTA and BAPTA. First, the cells were pre-treated with 1.5 mM of EGTA for three minutes before incubation with 20 µg/ml of lipoproteins for 48 hr, then analyzed by FISH. EGTA reduced by 3-fold the OX-LDL induced trisomy 21 (Figure 8A), and tetrasomy 21 and 12 (Figure 8B). The change in mitotic index between EGTA-treated cells with or without lipids (e.g. LDL) was not significant (107±29 vs. 81±42, p = 0.77, respectively).

hTERT-HME1 cells were also pre-incubated with another Ca++ chelator, BAPTA for three minutes and then co-treated with lipids for 48 hr. The results showed a statistically significant BAPTA-dependent reduction in OX-LDL-induced trisomy 21 and 12 (Figure 8C), and a borderline decrease in trisomy 21 in LDL-treated samples (p = 0.065; Figure 8C). Quantification and comparison of mitotic indices in BAPTA-treated hTERT cells with or without lipids (e.g., LDL) revealed no significant difference in number of metaphases per slide (67±18 vs. 69±37, p = 0.92, respectively), indicating that extracellular calcium chelation does not affect the progression of the cells through the cell cycle.

#### Cholesterol treatment does not cause DNA damage

Aneuploidy is characteristic of transformed cells and has been hypothesized to play an essential role in carcinogenesis [92]. Radiation, DNA-damaging chemicals, or genetic mutations, for example affecting mis-match repair, can lead to double strand breaks in genomic DNA [93], which has been shown to lead to chromosome mis-segregation and aneuploidy. However, we found no increase in double strand breaks as assessed by 53 BP1 immunocytochemistry after 48 hr treatment of hTERT cells with water-soluble cholesterol (Figure 9).

#### Cholesterol disrupts the mitotic spindle

There are many mechanisms by which genes or agents can induce chromosome mis-segregation [94]. For example, in Aß-treated cells and Xenopus egg extracts, the mitotic spindles become shortened and bent and the DNA becomes delocalized [24]. We treated hTERT-HME1 cells with 4 µg/ml WsCh for 24 hr and analyzed the mitotic spindles after staining with α-tubulin and γ-tubulin antibodies. As shown in Figure 10, cholesterol induced a multitude (30%) of aberrant mitotic/microtubule structures with disarrayed microtubules and displaced DNA being particularly prominent in mitotic structures.

## Discussion

We report a set of findings that reveal a novel cholesterol-dependent cell cycle defect that may be involved in the pathogenesis of Nieman-Pick C1 Disease, Alzheimer’s Disease and cardiovascular disease/atherosclerosis. Specifically: 1) Medium-term exposure to dietary cholesterol induces chromosome mis-segregation in peripheral tissues of young wild-type mice, 2) Disturbance in intracellular cholesterol homeostasis and obstructed cholesterol trafficking to the plasma membrane in NPC-1 patients is associated with an 4 to 6-fold increase in the proportion of trisomy 21 fibroblasts, neurons and glia, 3) Atherogenic lipoproteins (i.e., LDL, OX-LDL) and cholesterol, but much less so the protective HDL, induce chromosome mis-segregation, including trisomy HSA12, HSA21, and HSA7 and MMU16 in primary human and mouse cells *in vitro*, 4) Lipoproteins apparently require a functional LDL receptor but not the presence of the *APP* gene to exert their aneugenic effect, 5) Cholesterol exposure disrupts the mitotic spindle and induces disarrayed microtubules and displaced DNA, and 6) Reduction in either plasma membrane fluidity or Ca++ homeostasis decreases lipoprotein/cholesterol-induced aneuploidy *in vitro.*

### Aneugenic Effect of Lipoproteins and Cholesterol: Implications for AD and NPC

The present data complement and extend previous work showing that up 30% of AD human and mouse neurons and other cells are aneuploid due to either environmental or complex genetic influences. Although cholesterol and other aneugens, such as mutant *PS* or *APP* genes or the Aß peptide, affect all chromosomes in cultured cells, the lack of selection against trisomy 21 neurons probably accounts for their preponderance in AD and NPC-1 brain. The finding that LDL/cholesterol induces chromosome mis-segregation in the absence of *APP* indicates that the aneugenic pathways initiated by Aß and LDL are, at least in their early steps, separate and independent. While our analyses of actively dividing peripheral cells and precursor brain cells were limited to a few human and only one validated mouse chromosome probe, a comprehensive investigation of other peripheral and brain cells (i.e., neurons and glia) is warranted to fully understand the rate and type of chromosome instability in relation to changed cholesterol homeostasis.

Although there is some indication that aneuploid neurons develop normally in human brains, possibly increasing neuronal diversity [17], [95], the large number of aneuploid neurons in AD and NPC must arise from mitosis, either from disease-related neurogenesis, from reactivation of the cell cycle in normally-post-mitotic neurons, or both. A number of studies reported neuronal aneuploidy and tetraploidy of several chromosomes in the human brains diagnosed with AD [17], [18], [20], [21]. Here we did not observe a cholesterol-dependent increase in tetraploid cells (i.e., cells that had duplicated every chromosome, but failed to divide, or had not yet divided), but mitosis specific epitopes have been observed in a small number of neurons in AD and NPC brain or in mouse models of AD, and have been interpreted as indicative of such reactivation of the cell cycle and the consequent development of aneuploid neurons [25], [27], [28], [53]–[64]. However, the frequency of such tetraploid (4 n) neurons in AD is certainly less than 10% of the frequency of aneuploidy cells (with DNA content between 2 n and 4 n) [21], and some researchers report that the number of tetraploid cells is actually the same in brains from AD and control individuals and are exclusively non-neuronal [96]. Nevertheless, if neurons are induced to re-enter the cell cycle in response to neurodegenerative disease, the resulting mitosis and cell division might well be abnormal and result in the observed increase in aneuploidy, rather than tetraploid neurons.

While we failed to observe a change in ploidy in the brains of mice fed a high cholesterol diet, possibly due to the 1) length of dietary exposure, 2) reduced neurogenesis, 3) young age of the mice, and 4) low or nonexistent exchange of cholesterol and its metabolites across the intact blood-brain barrier (BBB), a peripheral induction of trisomy 16 in mitotically active tissues may be an initiating event that precedes the induction of brain aneuploidy once the extracerebral damage reaches a critical point and the BBB is compromised.

In conclusion, AD and NPC1 exhibit cell cycle abnormalities, to which excess lipoproteins, particularly LDL, can contribute and lead to the chromosome mis-segregation, aneuploidy and neurodegeneration observed in these diseases. Furthermore, the fact that aneuploid neurons also characterize other cognitive brain diseases, such as Frontotemporal Dementia [97] (also Granic et al., unpublished), Down syndrome and Ataxia-Telangiectasia [20], suggests that chromosome mis-segregation and the development of aneuploidy neurons may be a general feature of neurodegeneration.

### Chromosome Instability in Atherosclerosis: Implications for AD

Several studies have reported the hyperproliferation of aneuploid smooth muscle cells within atherosclerotic plaques [45]–[52], which our data suggest may be induced by elevated LDL-cholesterol. Specifically, during the initiation of atherosclerosis, a single smooth muscle cell that, as a consequence of aneuploidy, suffers altered gene regulation may lose checkpoint control or become more responsive to autocrine or paracrine growth factors (for example by becoming trisomy for chromosome 7, which encodes the PDGF receptor) and then hyper-proliferating to generate the observed thickening of the vascular wall. The fact that aneuploid cells are prone to apoptosis fits this model well, for apoptosis of smooth muscle cells is thought to be an essential contributor to the formation of atherosclerotic plaques [98]. Cholesterol induced aneuploidy would also be expected to initiate atherosclerosis in the brain, which can lead to the reduced blood flow and the development of ‘vascular dementia’ often seen AD patients [40]–[43], [99], [100].

Furthermore, the fact that inheritance of the *ApoE ε4* allele is the strongest known risk factor for developing sporadic AD other than age [101] (i.e., ApoE being the major cholesterol carrier protein in the brain [102]), also strengthens the potential significance of lipoprotein/cholesterol-induced aneuploidy for the pathogenesis of AD. LDL and ApoE4 also promote atherosclerosis [103], which is characterized by hyperproliferation of aneuploid cells, as discussed above [45]–[52], extending the connection between lipid-induced aneuploidy and both CVD and AD. Finally, the fact that Aß causes chromosome mis-segregation and aneuploidy [23] and that atherosclerosis arises in the AD brain and in mouse models of AD [40], [104] suggests that Aß itself may trigger the mis-segregation event that initiates the atherogenic process in AD.

### Mechanisms by which Aneuploidy may Contribute to Neurodegeneration in AD

There are several ways in which cholesterol (or Aß)-induced aneuploidy may contribute to cognitive decline in AD and related disorders. The most straightforward mechanism would be that aneuploid cells, with their altered genetic complements and thus altered gene expression are inherently prone to apoptosis/degeneration, as has been shown in many experimental systems [21], [105]–[107]. Indeed, as discussed above, the specific loss of (especially of non-trisomy 21) aneuploid neurons accounts for 90% of neurodegeneration in AD [9]–[23]. When the aneuploid neuronal progenitors and neurons harbor trisomy 21 or 16 and accumulate in the brains of AD patients and FAD transgenic mice respectively, overexpression of *APP* and other chromosome 21/16 genes will occur. That such imbalance may further contribute to disease onset and progression is evident in individuals harboring trisomy 21 mosaicism [25], [34]–[37] or an *APP* gene duplication [32], [33]. Similarly, the reduced neurogenesis and reduced neuronal number in Down syndrome [108], may result from the constitutive over expression of *APP* and production of Aß and the consequent induction of the cell cycle defect of chromosome mis-segregation.

Another possibility supported by the data, is that, like Aß, cholesterol disrupts chromosome segregation through inhibition of normal MT function. In addition to inducing defective neurogenesis as we demonstrate above, MT dysfunction could also affect neuronal plasticity. For example, we have found that Aß causes chromosome mis-segregation directly by binding to and inhibiting certain MT-dependent kinesin family motor proteins that are essential for mitotic spindle function [24]. These enzymes are also present in neurons, where they direct plasma neurotransmitter and neutrophin receptor localization and function at the plasma membrane [Ari et al. unpublished data]. As altered cholesterol homeostasis also disrupts microtubule structure and function, it could similarly affect receptor localization.

Finally, the counteracting effect of ethanol on lipid-induced chromosomal instability *in vitro* supports the hypothesis that the chromosome mis-segregation induced by lipoproteins is attributable to the increased membrane rigidity induced by cholesterol. We hypothesize that membrane stiffness upon cholesterol exposure may be restored by the fluidizing action of ethanol, making the cell less prone to mitotic errors. The fluidizing effect of ethanol on cell membranes is well established [83], [88], [89] and was proposed here as a possible mechanism for the reduction in aneuploidy in the cells co-incubated with cholesterol and alcohol, although no measurements of membrane fluidity were employed. Significantly, Aß peptide also increases membrane rigidity [109]. In the light of these data and the finding of aneuploid cells in AD brain and atherosclerotic plaques discussed above, it is interesting that moderate ethanol consumption has been linked to reduced risk of both CVD and AD [85]–[87], [110]. Prospective studies of microtubule stabilizers and/or ethanol or other modifiers of membrane fluidity may open new approaches to the prevention of neurodegenerative and cardiovascular disease.

### Conclusion

In this study we have presented *in vitro* and *in vivo* evidence that cholesterol dyshomeostasis, a common risk factor for NPC-1 and two age-related diseases (i.e., AD and atherosclerosis) may exert its pathogenic effect in part through disruption of the cell cycle. Specifically lipoprotein/cholesterol-induced chromosome instability/mis-segregation may be an underlying cytogenetic trait and part of the pathogenic pathway of multiple disorders, which opens new avenues for research and therapy.

## Materials and Methods

### Cell Line

#### hTERT-HME1 cells

The hTERT-HME1 cell line (Clontech) is a primary human mammary epithelial cell line that permanently expresses the telomerase reverse transcriptase and can thus divide indefinitely while retaining normal function, phenotype, and karyotype [111], [112]. The cell line was cultured in supplemented Mammary Epithelium Basal Medium (MEBM, Lonza) as described elsewhere [23].

### Primary Cells

#### HASMC

Human Primary Aortic Smooth Muscle Cells (HASMC) were isolated from healthy human aorta and cryopreserved as secondary culture at the density >5×10^5^ cells per vial (ScienCell Research Laboratories). The cells were maintained in the Smooth Muscle Cell Medium according to manufacturer’s specifications (SMCM; ScienCell Research Laboratories). Cells are subcultured when they were 90% confluent in 2 µg/ml poly-L-lysine (Sigma)-coated cell culture dishes.

#### Human fibroblasts

LDL receptor-negative human skin fibroblasts harboring two mutations, *C240-F* and *Y160-ter*, which cause a severe form of familial hypercholesterolemia (FH), fibroblasts with functional LDL receptor, four different human fibroblasts harboring *NPC1* mutations and four age-matched controls were obtained from Coriell Cell Repositories and cultured according to their recommended protocol.

#### Generation and maintenance of mNPCs

Mouse neurosphere cultures and derived neuronal precursor cells were established from non-transgenic prenatal brains (E17–E18) following a modified protocol by [77] and maintained according to [113].

#### Mouse splenocytes and brain cells

Mouse spleens and brains were harvested and processed to yield a cell suspension and prepared for karyotype and FISH analysis according to procedures previously described [22], [23].

#### Human brain cells

Frozen samples of frontal cortices from patients with the Niemann-Pick type C1 disease (age 20.88±8.64) and age, gender and race matched control brain tissues (age 17.26±11.07) were obtained from the NICHD Brain and Tissue Bank for Developmental Disorders at the University of Maryland (Baltimore, MD). Cell suspensions were prepared as previously described for mouse brains [23] and processed for FISH. Filipin and NeuN co-immunolabeling revealed significantly more cholesterol staining in the intracellular compartment in NPC neurons compared to controls.

### Mice, Feeding and Diets

Housing of and procedures involving all animals, including the time-pregnant C57BL/6 mice were approved by the USF Institutional Animal Care and Use Committee.

#### Wild-type mice

Eight one-month-old nontransgenic C57BL/6 mice (4 males and 4 females) (Jackson Lab) were caged in standard housing in pairs, and were fed *ad libitum* for 12 weeks with either regular chow, or high cholesterol diet (Harlan Laboratories) with free excess to fresh water. Food intake and weight gain were monitored and recorded weekly. The mice were anesthetized with i.p. injection of sodium pentobarbital (50 mg/kg weight), and the tissues harvested and inspected for pathological changes. Mice from dietary intervention were transcardially perfused with 0.9% saline before internal organs of interest were removed.

#### Diets

Regular mouse chow (2018 Tekland Global 18% Protein Rodent Diet, Harlan Laboratories) contains 5% crude plant-based oil (fat), 0% cholesterol, and supplies 3.3 Kcal/g of Digestible Energy (DE). Custom-made high cholesterol diet (TD.95286, Harlan) consists of 21% milk fat (fat and 0.05% cholesterol), 1% of cholesterol, and provides 4.5 Kcal/g of DE.

#### APPKO mice

Mice lacking an *APP* gene and their non-transgenic littermates were 3–4 months of age (Jackson Labs) and had C57BL/6 background strain. The spleens were used for primary cell cultures and lipoprotein/cholesterol treatments.

### Reagents and Kits

#### Lipoproteins

Human oxidized LDL (protein concentration 2 mg/ml), human LDL (protein concentration 5 mg/ml) and human HDL (protein concentration 10 mg/ml) were purchased from Biomedical Technologies Inc. Water-Soluble Cholesterol (WsCh) (Sigma; 31.8 mg of pure cholesterol in 0.6 g total weight of solid, balanced in methyl-beta-cyclodextrin) was resuspended in sterile water.

#### Other reagents, kits and DNA probes

EGTA (Sigma) and BAPTA (Calbiochem) were used as Ca++ buffering reagent at 1.5 mM and 1 mM, respectively. hTERT-HM1 cells are cultured in medium that has 2.65 mM calcium. Absolute (without benzene) ethyl alcohol (Sigma-Aldrich) was prepared as a sterile aqueous 1 M solution and aliquoted. Filipin complex (Sigma) was dissolved in DMSO (Sigma) and stored at −20°C in small aliquots and protected from light. Giemsa stain (Gibco/Invitrogen) was diluted freshly in 1X GURR buffer (GibcoBRL Life Technologies) to stain metaphase chromosomes. Mouse anti-neuronal nuclei (NeuN) AlexaFluor® antibody (Millipore) and VectaShield with DAPI (Vector Laboratories) was used to label neurons and nuclei, respectively. Anti-53BP1 rabbit polyclonal (Abcam) and AlexaFluor 488 goat-anti-rabbit antibody (Invitrogen, Molecular Probes) were used to detect DNA double-strand breaks (DSBs) foci, anti-α-tubulin (clone B-5-1-2, Sigma) and AlexaFluor 488 goat-anti-mouse antibodies (Invitrogen) were used to stain mitotic spindles, and anti-γ-tubulin (Sigma) and AlexaFluor 594 goat-anti-rabbit antibodies (Invitrogen) were utilized to detect centrosomes. Nick Translation Kit (Roche) and Spectrum Green or Spectrum Orange dUTPs (Vysis) were used to generate the chromosome 16 BAC probe as previously described [22]. Ethanol Assay Kits (BioVision) and BioTek Synergy HT micro-plate reader (BioTek Instruments) for colorimetric assay (O.D. 570 nm), and Gen5™ (BioTek Instruments) data analysis software were used to measure ethanol concentration in the media at baseline, after 24 and 48 hr of exposure following manufacturer’s instructions. Oil-Red-O staining kit (American MasterTech) was used to stain for neutral lipids accumulation in mouse hepatocytes. Fluorescently labeled DNA probes detecting human chromosome 21, 12, and, 18, 14 and 7 (LSI® TEL/AML1 ES Dual Color Translocation Probe, LSI® IGH (14q32)/MALT1(18q21) Dual Color Dual Fusion Probe and LSI® D7S522 (7q31) SpectrumOrange ™/CEP® 7 SpectrumGreen™, respectively) were purchased from Abbott, Vysis.

### In Vitro Incubation with Lipoproteins/Cholesterol

Two days prior to treatment/incubation, freshly passaged hTERT cells (1–3×10^5^ cells/2 ml) were plated in 100 mm tissue culture dishes in MEBM to assure 60%–70% confluency on the day of the treatment. The cells were then incubated for 48 hr at the concentration of 20 µg/ml of lipoproteins or 2 and 4 µg/ml of WsCh.

Seven parallel experiments were used to assess the effect of lipoproteins on chromosome segregation (i.e., karyotype analysis and chromosome specific aneuploidy) and six separate experiments to test the aneugenic effect of cholesterol. The same cell passages were used for the set of parallel experiments involving Ca++ chelating reagents, BAPTA or EGTA performed as in [23].

Similarly, two days before lipid treatment, freshly passaged HASMC, human fibroblasts with and without a functional LDL receptor or hTERT-HME1 cells were seeded onto pre-coated (2 µg/ml of poly-L-lysine, Sigma) single glass chamber slides (8.6 cm^2^ growth surface/well) (BD Bioscience) at the density of 100,000 and 200,000 cells per slide respectively. After 24 or 48 hr of lipid/cholesterol incubation, the cells were rinsed twice with 1X PBS without Ca and Mg (Cellgro), immediately fixed with either cold 3∶1 anhydrous methanol and acetic acid fixative or methanol, and incubated at −20°C for 30 min.

Mouse non-transgenic and *APPKO* spleen cells were similarly incubated with lipoproteins for 48 hr, harvested and fixed for FISH analysis as described [22], [23].

### In Vitro Ethanol and Lipoprotein/Cholesterol Incubation

To assure an exact and consistent ethanol (EtOH) concentration in treated cells, we used a modified closed chamber system protocol by Adickes et al. [114]. Prior to ethanol and lipoproteins co-treatments, several pilot experiments were conducted to examine the viability of hTERT-HME1 cells in the closed chamber, and to establish cytotoxicity of 25 mM and 50 mM of EtOH for 6, 12 and 24 hr on cell morphology, proliferation and survival (Trypan Blue dye exclusion). Based on these results, triplicate experiments were conducted with 25 mM EtOH for 24 hr followed the next day by lipoproteins (20 µg/ml) and WsCh (4 µg/ml) and EtOH co-incubation for 48 hr. A small aliquot of EtOH containing media from two samples was collected at the baseline, 24 and 48 hr to measure alcohol concentration using colorimetric assay (Ethanol Assay Kit, BioVision). Lastly, the cells were harvested and fixed for FISH analysis.

### Metaphase Chromosome Analysis

After colcemid treatment, hTERT-HME1 cells were harvested and the metaphase chromosomes counted as described [22], [23]. Genus 2.81® software was used for chromosome analysis (Applied Imaging). At least 40–45 metaphases were analyzed per each sample.

### Fluorescence in situ Hybridization (FISH)

Prior to hybridization, all slides were aged at room temperature followed by hybridization procedures recommended by Abbott, Vysis and by protocols and mouse and human probes described above and previously [22], [23]. Hybridizations were done according to manufacturer’s instructions (Abbott, Vysis) for the selected probes in a HyBrite hybridization chamber (Vysis) followed by DAPI II (Abbott, Vysis) or Vectashield with DAPI (Vector Laboratories) counterstain.

### FISH Followed by Immunocytochemistry of NPC1 Brain Cells

To assess neuronal versus glial cell aneuploidy in NPC1 and control brains, we used a combination of FISH and immunocytochemistry [23]. Briefly, immediately after FISH, brain cells were incubated in 1XPBS for 10 min, blocked in 10% goat serum/0.1% Triton X-100 PBS solution for 1 hr followed by overnight incubation in conjugated Ms X Neuronal Nuclei AlexaFluor 488 (Millipore) 1∶100 antibody prepared in 1% BSA/0.1% Triton X-100 1XPBS. After final washes in 1XPBS, the cells were stained with DAPI II counterstain.

### Immunocytochemical Quantification of Double Strand Breaks

Double strand breaks, an indication of DNA damage were identified in cholesterol-treated hTERT-HME1 cells by immunostaining for 53 BP1-containing foci. First, cell pellets were fixed in 70% cold ethanol, dropped onto Frosted slides (Fisher Scientific) and air-dried. Second, immediately after fixation in 4% paraformaldehyde for 10 min, and three 1XPBS washes, the cells were permeabilized in 0.2% Triton X-100 1XPBS solution for 10 min, blocked in 5% BSA 1XPBS/0.1% Tween-20 for 30 min, and incubated in 1% BSA 1XPBS/0.1% Tween-20 solution containing anti-53 BP1 rabbit polyclonal antibody (Abcam), 1∶400 for 1 hr followed by AlexaFluor 488 goat-anti-rabbit antibody (Invitrogen), 1∶300 incubation for the same time period. After several 1XPBS washes, the cells were stained with DAPI II (Vector).

### Immunocytochemistry for Spindle Analysis

Upon fixation in methanol, cholesterol treated hTERT-HME1 cells were co-immunostained for mitotic spindles and centrosomes with anti-α-tubulin (Sigma) and anti-γ-tubulin antibodies (Sigma) 1∶1000, followed by AlexaFluor 488 goat-anti-mouse and AlexaFluor 594 goat-anti-rabbit secondary antibodies (Invitrogen, Molecular Probes), 1∶2000 incubation, respectively and Vectashield mounting medium with DAPI staining (Vector) as described [23].

### Oil-Red-O Staining

Cell suspension of snap-frozen livers (2 from mice fed a regular and 2 from mice fed a high cholesterol diet) were made in 1XPBS, dropped onto pre-wetted slides, and stained with the Oil-Red-O staining kit following manufacturer’s procedures for frozen tissues with slight modifications (American MasterTech). Hematoxylin stained single cells and cell aggregates (blue) were evaluated under phase microscopy (Zeiss Imager M1) for the presence of lipid droplets (red).

### Image Acquisition and Analysis

FISH hybridization signals were analyzed according to Abbott/Vysis guidelines as described in [22], [23] using a Nikon Eclipse E1000 fluorescence microscope with a 4912 CCIR high performance COHU CCD Camera and Genus 2.81® software (Applied Imaging) or a Zeiss Imager M1 microscope with a CV-M4+CL high resolution camera and Axiovision4.6 software (Zeiss) or a Zeiss Axiovert 135 with AxioCam HRc (Zeiss). The frequencies of nuclei containing 0, 1, 2 or 3 53 BP1 signals were recorded in treated and control cells, and mitotic spindle phenotypes were analyzed as described [22]. On average, between 700 and 1,000 interphases per each sample/treatment were scored for aneuploidy, and 400 NeuN positive and NeuN negative NPC1 brain cells were analyzed for DNA hybridization signals. More than 450–600 cells for each of three samples of both WsCh-treated and control were assessed for 53 BP1 foci, and more than 45–80 spindles from each of four samples of both treated and control cells were analyzed for spindle abnormalities.

Hematoxylin and Oil-Red-O stained mouse hepatocytes and cell aggregates from mice fed a regular and high cholesterol diet were compared and contrasted for amount of red staining under phase microscopy (Zeiss).

### Statistical Analysis

Paired Student’s T-test was used to compare the aneuploidy induced by various lipoproteins with or without Ca++ chelating reagents and ethanol in different cell lines across multiple experiments and in spleen and brain cells of mice fed different diets, and to compare the percentages of nuclei containing 1 to 3 DSBs foci and the levels of abnormal spindle events between treated and untreated cells. Independent T-test was used to compare the aneuploidy between NPC1 fibroblasts and NPC1 brain cells and appropriate controls. Three to 7 treatments with lipids/cholesterol for each cell line, and triplicate experiments with chelators and ethanol were conducted and scored for aneuploidy. Four to 5 primary NPC1 cells sources and controls and at least 6–12 mice were analyzed for each graph.

#### Ethics statement

All experiments were carried out in accord with the University of South Florida guidelines for examination of human tissue and the ethical treatment of animals. Because the only human tissues were cell lines and autopsy brain tissue obtained from biorepositories, and we were not provided any identifying information, the experiments are exempt. The animal work was carried out under the USF IACUC oversight and approval (M3785; Promoters and Inhibitors of Alzheimer’s Disease Pathology, Colony and R3138 and R3786 (different times); Promoters and Inhibitors of Alzheimer’s Disease Pathology, Research).

## Supporting Information

Figure S1 **25 mM of Ethanol (EtOH) does not induce aneuploidy within 48 hr.** hTERT-HME1 cells were treated with 25 mM EtOH for 24 and 48 hr in a closed chamber system. (A) No induction of trisomy 21 (or trisomy 12) was observed, but there was an increase in tetrasomy 21 (presumably cells in G2), within 24 hr (B). After further incubation in EtOH-containing media, levels of such tetrasomy 21 cells returned to background (C–D).
(TIF)
