# Supplementary figures and images for: Mitotic Spindle Defects and Chromosome Mis-Segregation Induced by LDL/Cholesterol—Implications for Niemann-Pick C1, Alzheimer’s Disease, and Atherosclerosis
Source: PLoS One. 2013 Apr 12;8(4):e60718. doi: 10.1371/journal.pone.0060718 (PMC3625184; doi:10.1371/journal.pone.0060718)

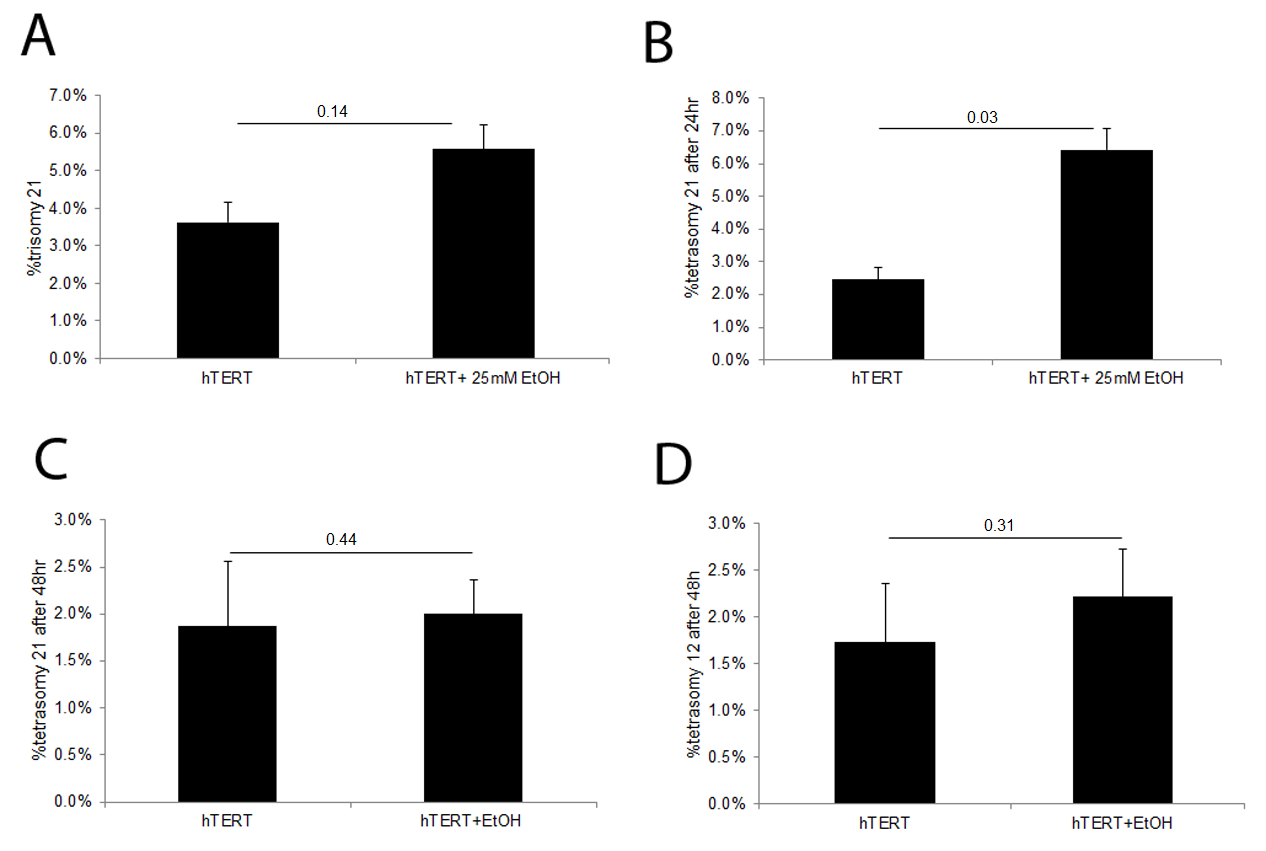

Supplement: Figure S1 — 25 mM of Ethanol (EtOH) does not induce aneuploidy within 48 hr. hTERT-HME1 cells were treated with 25 mM EtOH for 24 and 48 hr in a closed chamber system. (A) No induction of trisomy 21 (or trisomy 12) was observed, but there was an increase in tetrasomy 21 (presumably cells in G2), within 24 hr (B). After further incubation in EtOH-containing media, levels of such tetrasomy 21 cells returned to background (C–D). (TIF) [file pone.0060718.s001.tif]
